# S18 family of mitochondrial ribosomal proteins: evolutionary history and Gly132 polymorphism in colon carcinoma

**DOI:** 10.18632/oncotarget.10957

**Published:** 2016-07-30

**Authors:** Muhammad Mushtaq, Raja Hashim Ali, Vladimir Kashuba, George Klein, Elena Kashuba

**Affiliations:** ^1^ Department of Microbiology, Tumor and Cell Biology (MTC), Karolinska Institute, Stockholm, S-17177, Sweden; ^2^ KTH Royal Institute of Technology, Science for Life Laboratory, School of Computer Science and Communication, Solna, SE-17 177, Sweden; ^3^ Institute of Molecular Biology and Genetics of NASU, Kyiv, 03680, Ukraine; ^4^ R.E. Kavetsky Institute of Experimental Pathology, Oncology and Radiobiology, NASU, Kyiv, 03022, Ukraine

**Keywords:** mitochondrial ribosomal proteins, MRPS18 family, phylogenetic analysis, evolutionary trace analysis, genetic polymorphism

## Abstract

S18 family of mitochondrial ribosomal proteins (MRPS18, S18) consists of three members, S18-1 to −3. Earlier, we found that overexpression of S18-2 protein resulted in immortalization and eventual transformation of primary rat fibroblasts. The S18-1 and −3 have not exhibited such abilities. To understand the differences in protein properties, the evolutionary history of S18 family was analyzed. The S18-3, followed by S18-1 and S18-2 emerged as a result of ancient gene duplication in the root of eukaryotic species tree, followed by two metazoan-specific gene duplications. However, the most conserved metazoan S18 homolog is the S18-1; it shares the most sequence similarity with S18 proteins of bacteria and of other eukaryotic clades. Evolutionarily conserved residues of S18 proteins were analyzed in various cancers. S18-2 is mutated at a higher rate, compared with S18-1 and −3 proteins. Moreover, the evolutionarily conserved residue, Gly132 of S18-2, shows genetic polymorphism in colon adenocarcinomas that was confirmed by direct DNA sequencing.

Concluding, S18 family represents the yet unexplored important mitochondrial ribosomal proteins.

## INTRODUCTION

Mitochondrial ribosomal proteins (MRPs) are encoded by nuclear genomic DNA, MRPs are important structural constituents of mitoribosome, a key component of mitochondrial translation machinery. Mammalian 55S mitoribosome is composed of two parts: 28S small and 39S large subunits. The 28S subunit is involved in mRNA binding [1] and decoding, whereas 39S subunit assists the mitoribosome in catalysis of peptidyl transferase reactions [2]. During the evolution of the 55S mammalian mitoribosome, the ancestral mitoribosome (70S) underwent key structural alterations. Mammalian mitoribosome contains more proteins than the bacterial ribosome; therefore, 55S ribosomes are larger than bacterial ribosomes despite the loss of approximately half of their RNA [3].

Functions of a few MRPs have been characterized and are central to oxidative phosphorylation (OXPHOS) pathways [4]. Several MRP genes have been mapped to loci associated with disorders consistent with defective oxidative phosphorylation, such as multiple mitochondrial dysfunctions, Leigh syndrome, and nonsyndromic hearing loss [5].

MRPS18 (termed S18 here) proteins are grouped in a family, consisting of three homologs in Metazoa (S18-1–3) and one homolog in other cellular organisms. Three S18 proteins differ remarkably in size, ranging from 11.7 to 27 kDa. The human *S18-1* gene lies on chromosome 4q21.23, whereas *S18-2* is located on chromosome 6p21.3 and *S18-3* was mapped to chromosome 6p21.1 [6]. One of S18 proteins, the S18-2, plays an important role in cell immortalization by binding to retinoblastoma (RB) protein and advancing the cell cycle through G_1_ to S phase [7]. The overexpression of S18-2 in rat fibroblasts led to their immortalization [8, 9] or transformation [10].

However, no other data were reported on the physiological function of S18 family proteins. While the evolution of whole proteome of mitoribosome was studied [5], a comprehensive evolutionary analysis of S18 proteins has not been performed. Recently, the crystallographic structure of mammalian 55S mitoribosome is resolved at a resolution of 3.8Å, using cryo-electron microscopy [11]. The structure suggests that three S18 homologs are localized to three distinct sites in mammalian mitoribosome, contrary to the previous assumption that all of them reside on a single site in a mutually exclusive fashion [1, 4, 12].

The present study is devoted to two aims: (i) to gain insight into the evolution of S18 protein family and (ii) to create a link between the knowledge obtained from evolutionary analysis of S18 proteins and cancer development in humans. Here we focused on origins, evolutionary patterns and phylogeny of S18 family proteins in eukaryotic and bacterial clades, and also analyzed the mutations of evolutionarily conserved residues in different types of cancer.

## RESULTS

### S18 proteins possess a single Ribosomal_S18 domain in eukaryotes, except for S18-3 in catarrhines

The *S18* gene was found in several taxonomic clades of bacteria and eukaryotes, from Unikonta (represented by *Dictyostelium discoideum*) to Metazoa (represented by *Amphimedon queenslandica*), using the literature data. In this way, a set of 19 bacterial species representing all major taxonomic clades of bacteria was identified (Supplementary Table S1). Similarly, 59 eukaryotic species ranging from Amoebozoa to primates were identified (Supplementary Table S2). In total, 118 homologs of S18 protein in eukaryotes and 19 homologs in bacteria were used to perform comprehensive phylogenetic analysis of the S18 family.

**Figure 1:**
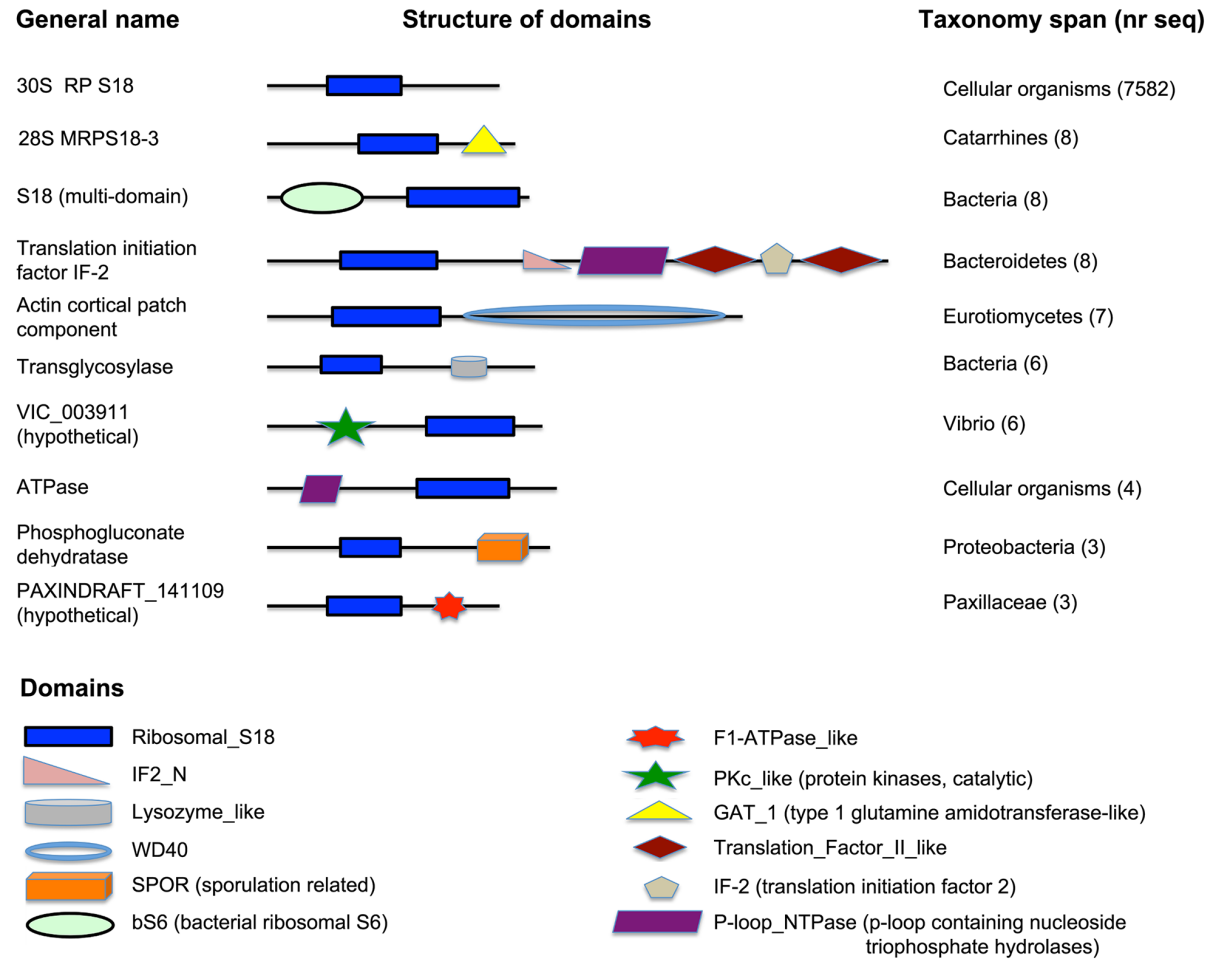
Domain architecture of S18 proteins A schematic representation of domain architecture of proteins, containing the Ribosomal_S18 domain. Notice the domain architecture diversity in bacterial S18, universal architecture of eukaryotic proteins, containing the Ribosomal_S18 domain, and the unique architecture of S18-3 in catarrhines.

All S18 proteins are characterized by the presence of a Ribosomal_S18 domain, a conserved sequence of approximately 152 amino acids across all species. The bacterial S18 is diverse in terms of domain architecture. The Ribosomal_S18 domain-only containing protein is, by far, the most observed architecture in bacteria and in eukaryotes (shown on Figure 1), suggesting probable domain fusion (or domain insertion) event in bacteria.

However, there is a lineage-specific protein fusion event observed in S18-3 of Catarrhini (old world monkeys, a group consisting of *Macaca* and *Papio*, etc.). The S18-3 protein consists of two domains, Ribosomal_S18 and GAT_1 (Type 1 glutamine amidotransferase-like), as shown in the second line on Figure 1. Proteins, consisting of only GAT_1 domain, and other proteins, consisting only of Ribosomal_S18 domain, are observed in all cellular organisms.

A local gene neighborhood (BLAST-based syntenic conservation in genomic content) Pattern between chromosomal regions containing *Homo sapiens* S18-3, *Macaca* S18-3 and *Papio* S18-3 showed three homologous regions. This supports the hypothesis that a possible gene fusion event in Catarrhini gave rise to multi-domained S18-3 protein.

### Conservation profile of S18 homologs

Another way to verify and quantify the selection pressure on S18 homologs is to determine the conservation profile of S18 homologs. There is only one extant S18 protein in most bacterial species. In contrast, there are three extant Metazoan homologs, which have evolved from a common ancestral gene in eukaryotes. The conservation profile was checked separately for probable S18 orthologs (genes related by speciation as their last common ancestor) in bacteria and for S18 homologs in Metazoa.

The conservation profile (Table 1) for bacteria suggests that S18 orthologs are highly conserved (around 50% sequence identity on average) across bacterial species, despite long divergence times on the species tree (Supplementary Figure S1). The Ribosomal_S18 domain, as expected, is sufficiently conserved throughout the evolution in bacteria (Supplementary Figure S2).

**Table 1:** The conservation profile of S18 in bacterial species*

	*Firmi cutes*	*Chloro flexi*	*Cyano bacteria*	*Deino coccus -Thermus*	*Actino bacteria*	*Gamma proteo bacteria*	*Beta proteo bacteria*	*Alpha proteo bacteria*	*Acido bacteria*	*Delta proteo bacteria*	*Epsilon proteo bacteria*	*Chla my diae*	*Chloro bi*	*Bactero idetes*	*Plancto mycetes*	*Spiro chaetes*	*Fuso bacteria*	*Aqui ficae*	*Ther mo togae*	Species Average Distance
*Firmicutes*	100	54,43	54,17	45,95	50,68	51,35	50	58,67	56,58	52,7	50,63	50	50	54,17	57,97	51,9	50	45,33	54,67	54,69
*Chloroflexi*	54,43	100	47,3	44,16	43,04	42,5	43,9	48,68	52,5	41,33	41,67	45,45	38,82	44,59	45,07	36,47	37,31	33,33	45,57	46,64
*Cyano bacteria*	54,17	47,3	100	59,46	50	50	51,35	47,22	52,78	49,28	43,24	50,7	48,65	43,84	46,38	43,24	46,77	40,54	49,32	51,28
*Deino coccus-Thermus*	45,95	44,16	59,46	100	47,3	49,33	40,26	39,19	50,67	48,57	46,75	51,39	37,66	46,58	40,58	38,96	50	31,58	47,37	48,20
*Actino bacteria*	50,68	43,04	50	47,3	100	38,82	37,84	45,83	41,67	43,66	47,37	50	35,9	43,84	42,25	42,86	40	37,84	44,59	46,50
*Gamma proteo bacteria*	51,35	42,5	50	49,33	38,82	100	44	42,47	42,47	53,52	41,56	47,22	40,51	47,95	43,66	44,87	36,36	36	41,33	47,05
*Beta proteo bacteria*	50	43,9	51,35	40,26	37,84	44	100	64,47	53,26	46,58	42,05	50	39,76	46,67	50,72	40,45	37,68	32,53	42,17	48,09
*Alpha proteo bacteria*	58,67	48,68	47,22	39,19	45,83	42,47	64,47	100	55,41	58,33	50	51,39	43,42	44,44	52,17	52,63	43,55	42,67	56,76	52,49
*Acido bacteria*	56,58	52,5	52,78	50,67	41,67	42,47	53,26	55,41	100	49,3	44,19	52,7	44,44	52,05	53,73	42,53	44,12	32,1	56,1	51,40
*Delta proteo bacteria*	52,7	41,33	49,28	48,57	43,66	53,52	46,58	58,33	49,3	100	69,33	59,46	46,67	59,42	52,17	52	49,18	40,85	54,93	54,07
*Epsilon proteo bacteria*	50,63	41,67	43,24	46,75	47,37	41,56	42,05	50	44,19	69,33	100	55,84	42,35	52	45,07	42,22	40,85	36,14	42,86	49,16
*Chlamydiae*	50	45,45	50,7	51,39	50	47,22	50	51,39	52,7	59,46	55,84	100	38,16	52,86	52,94	42,86	46,77	35,62	49,32	51,72
*Chlorobi*	50	38,82	48,65	37,66	35,9	40,51	39,76	43,42	44,44	46,67	42,35	38,16	100	54,67	45,07	38,37	46,38	35	43,21	45,74
*Bactero idetes*	54,17	44,59	43,84	46,58	43,84	47,95	46,67	44,44	52,05	59,42	52	52,86	54,67	100	52,17	41,33	48,39	38,67	51,35	51,32
*Plancto mycetes*	57,97	45,07	46,38	40,58	42,25	43,66	50,72	52,17	53,73	52,17	45,07	52,94	45,07	52,17	100	42,25	54,1	47,83	53,62	51,46
*Spiro chaetes*	51,9	36,47	43,24	38,96	42,86	44,87	40,45	52,63	42,53	52	42,22	42,86	38,37	41,33	42,25	100	51,39	33,73	42,86	46,36
*Fuso bacteria*	50	37,31	46,77	50	40	36,36	37,68	43,55	44,12	49,18	40,85	46,77	46,38	48,39	54,1	51,39	100	33,33	47,83	47,58
*Aquificae*	45,33	33,33	40,54	31,58	37,84	36	32,53	42,67	32,1	40,85	36,14	35,62	35	38,67	47,83	33,73	33,33	100	40,96	40,74
*Thermo togae*	54,67	45,57	49,32	47,37	44,59	41,33	42,17	56,76	56,1	54,93	42,86	49,32	43,21	51,35	53,62	42,86	47,83	40,96	100	50,78

* The conservation profile was measured in bacterial species for probable S18 orthologs on the basis of pairwise divergence time. At the end, average conservation of S18 was calculated among bacterial species indicating that S18 is highly conserved in bacterial species with average conservation of approximately 50%. Total average distance is 49,22.

**Figure 2:**
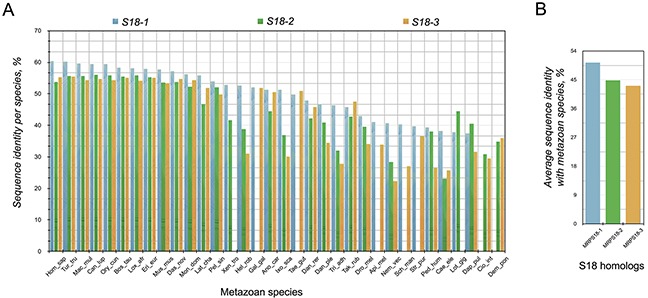
Percentage similarity between S18 orthologs of different species **A.** percentage sequence identity between different orthologs of S18 protein family in metazoan; **B.** average percentage sequence identity of the three members of S18 protein family in metazoa. S18-1 has, in general, the highest bars and is the most conserved homolog of the three S18 proteins.

However, there are differences in the conservation profiles of S18 homologs in Metazoa. The S18-1 protein in all species has, in general, a higher average species sequence identity compared with average species sequence identity of the S18-2 and S18-3 (Figure 2A). The S18-1 is also the most conserved of all homologs across metazoan species - on average 52% sequence identity as compared with 45% for S18-2 and 44% for S18-3 (Figure 2B). This depicts relaxation of selection pressure on the S18-2 and on S18-3. The S18-1 shows the same conservation profile on average as that of S18 orthologs in bacteria.

### Evolutionary trace analysis of S18 family of proteins

**Figure 3:**
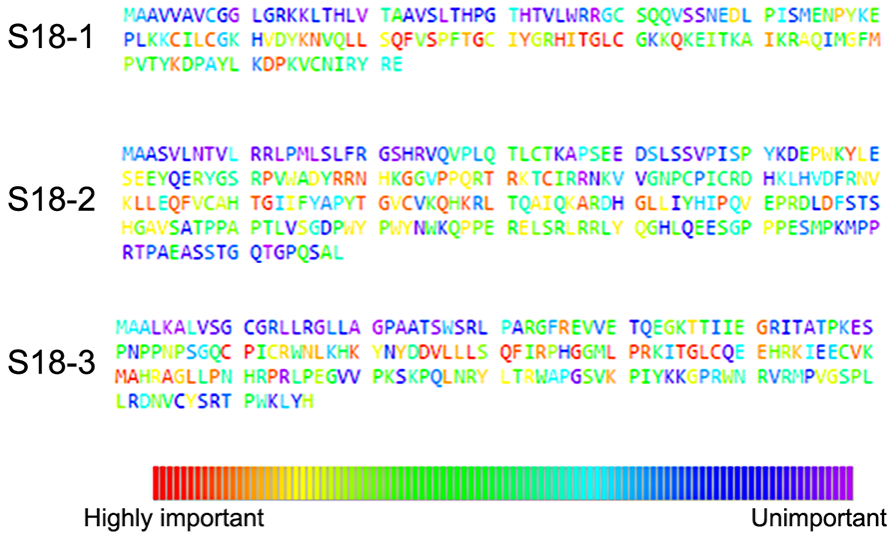
Evolutionary trace analysis of S18 family proteins The analysis was performed online at the ETA web server. The Uniport ID for S18-1, S18-2 and S18-3 were given to ETA web server, BLAST was performed and sequences were aligned by MUSCLE software. The results were presented as heat diagram which shows different grades of residues in terms of evolution conserved residues. The highly conserved residues are marked in red. The majority of the evolutionarily important residues are situated in the middle region of the three proteins.

Evolutionary trace analysis (ETA) was employed to determine the evolutionarily conserved residues in S18 homologs and map them on protein structure to identify important structural and functional properties of these conserved traces. The surface structures of S18 proteins were colored as heat diagram indicating red residues as evolutionary conserved/important amino acids (Supplementary Figure S3A). The evolutionarily important residues within the Ribosomal_S18 domain are presented as HMM-logos [13] (see Supplementary Figure S4). The details of all conserved amino acids in complete sequences of the S18-1, -2 and -3 are presented on Figure 3 and in Supplementary Table S3. The results from ETA show that most of the highly important residues are within the Ribosomal_S18 domain of S18 proteins. It was proposed, that all three mitochondrial S18 homologs are zinc-binding proteins. Three cysteine residues of S18-3 - Cys70, Cys73, and Cys108 and one Cys64 of MRPL10 participate in binding to zinc ion [11]. The ETA analysis shows that all residues that contribute to the binding of zinc ion are highly conserved, except the Cys73 of S18-3 (Supplementary Figure S3B).

### Phylogenetic analysis

Phylogenetic analysis of homologs of a certain gene reveals the evolutionary history and the probable gene duplication, loss and also the substitution patterns during vertical (tree-like) evolution from a single ancestral gene. In this regard, the bacterial and eukaryotic *S18* genes were separately analyzed.

**Figure 4:**
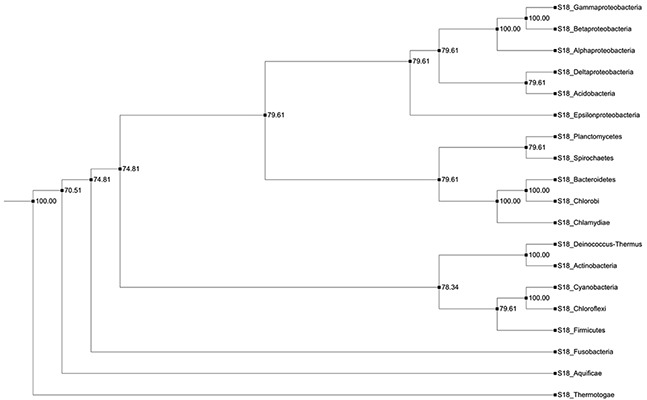
Consensus tree of bacterial S18 protein A cladogram representing the majority rule consensus tree of posterior of bacterial gene tree. All branches in the tree are well supported in the posterior.

The bacterial *S18* gene tree that was obtained, using JPrIMe-DLRS strongly follows the species tree, indicating no gene duplication or loss events in the nineteen bacterial species that were analyzed. Another result, supporting this tree, is the same gene tree topology that was obtained, using Bayesian statistics. The latter included i) the Maximum Likelihood (ML) state tree, ii) the Maximum a posteriori probability (MAP) tree (tree observed with highest frequency in the tree posterior (84%)), and iii) the majority-rule consensus tree (Figure 4). Hence, it may be concluded that bacterial *S18* gene is highly conserved and, probably, does not have any duplications or losses in bacteria.

**Figure 5:**
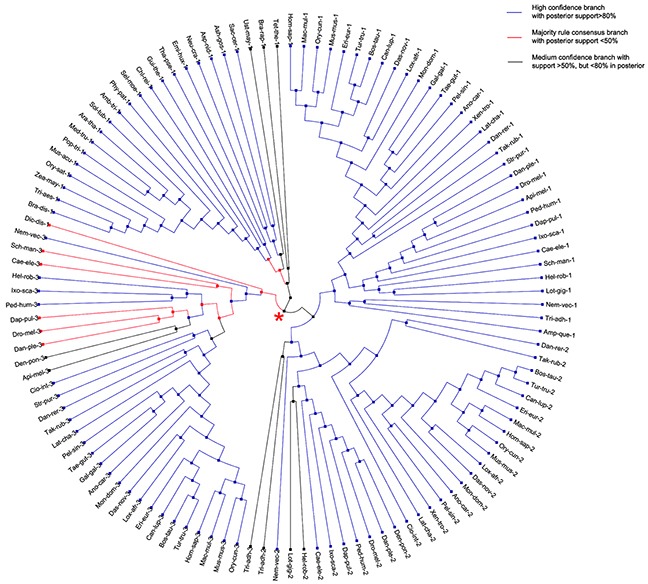
Consensus tree of eukaryotic S18 protein A circular cladogram representation of the majority rule consensus tree of posterior of eukaryotic gene tree. Branches are colored according to the amount of support in the posterior. Most branches are highly supported. The red star indicates the node at which the ancestral S18 gene gave rise to S18-3 and the parent of S18-1 and S18-2.

Phylogenetic analysis of eukaryotic *S18* genes, however, shows a different pattern to their bacterial homolog. While bacterial ancestral *S18* gene evolved without any gene duplications or losses (see above), its eukaryotes homologs underwent a couple of gene duplications and losses upon the evolution within eukaryotic species tree. After removing burning samples from the posterior, MAP tree and ML state tree (shown on Supplementary Figure S5) are the same for eukaryotic *S18* genes. However, the consensus tree of the posterior (shown on Figure 5) differs from MAP and ML state trees. Except for a few splits close to a root of the species tree, all splits are highly supported in tree posterior with posterior frequency greater than 80% (Supplementary Figure S6). The three homologs - *S18-1*, *S18-2* and *S18-3* - are Metazoan-specific and cannot be found in Fungi, Viridiplantae or Protists. Moreover, the phylogenetic gene tree reveals that the ancestral *S18* gene gave rise to *S18-3* and the parent of *S18-1* and *S18-2*, which can be seen at the parent node (shown with red star on Figure 5) in the eukaryotic *S18* gene tree.

### Ancient gene duplication was followed by three rounds of such events observed in metazoan during the evolution of eukaryotic S18

Another significant query investigated in this study is the number and placement of gene duplication events that gave rise to *S18-1*, *S18-2* and *S18-3* in Metazoa. For this purpose, eukaryotic S18 consensus gene tree (discussed in previous paragraph) was reconciled with the eukaryotic species tree.

**Figure 6:**
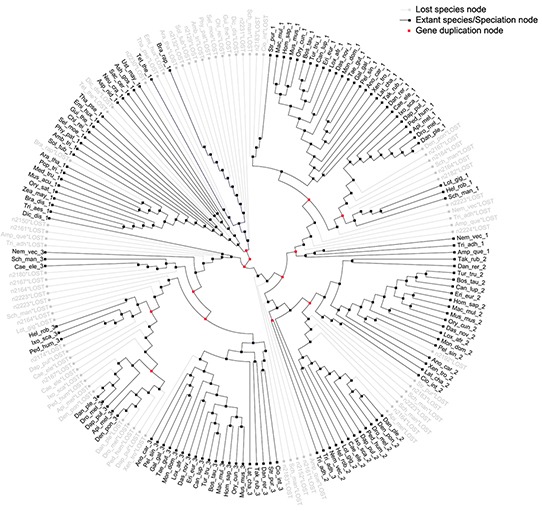
Most parsimonious reconciliation between eukaryotic S18 consensus gene tree and species tree A cladogram showing the duplications and losses in the most parsimonious reconciliation of eukaryotic S18 consensus gene tree and species tree. The tree contains thirteen duplications and seventy losses. Most branches, in particular, the metazoan branches have no duplications or losses. Duplications are shown with red nodes and grey branches represent losses.

The most parsimonious reconciliation (MPR) between the eukaryotic S18 consensus gene tree and eukaryotic species tree, determined from NOTUNG (see the circular cladogram on Figure 6) points to ancient gene duplication in the stem of the species tree. Therefore, the fungal *S18* genes are grouped with Protist and not with Metazoa in the gene tree.

**Figure 7:**
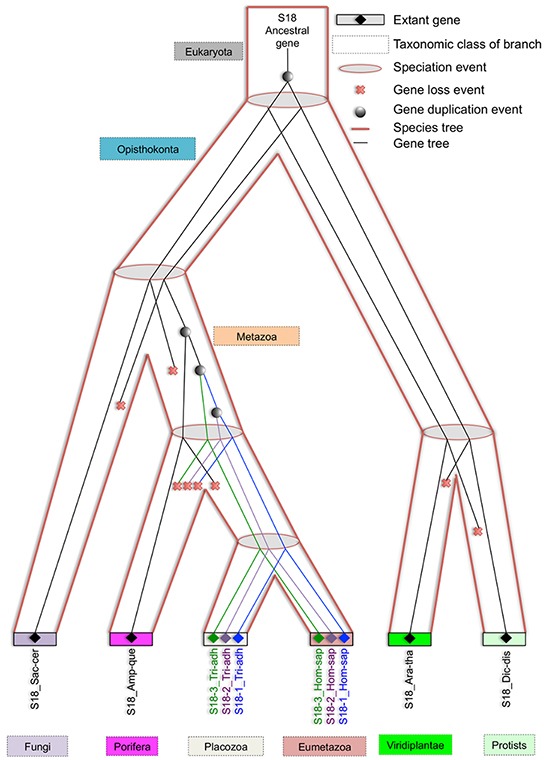
Evolving eukaryotic S18 consensus gene tree inside species tree An illustration of how eukaryotic S18 consensus gene tree is evolving inside the eukaryotic species tree from an ancestral S18 protein. An ancient gene duplication is proposed in the stem of species tree by the reconciliation. Also, three gene duplications are proposed in the metazoan branch, which gave rise to modern day S18-1, S18-2 and S18-3 proteins in placozoa and eumetazoa clades.

As shown on Figure 7, three rounds of duplication took place in metazoan lineage. One duplicate was lost in the branch, leading to a parent of Eumetazoa and Placozoa. The first round of duplication gave rise to *S18-3* and the parent of *S18-1* and *S18-2*. Another round of duplication resulted in *S18-1* and *S18-2*. The duplication pattern is also supported by the fact that *S18-2* and *S18-3* localized to the same chromosome of mammalian genome, for example, human chromosome 6. This indicates that the first duplication of the ancestral *S18* gene resulted in both daughter duplicates, being retained in the same chromosome. The second duplication event was followed by translocation of one gene to another chromosome.

### Mutational analysis of evolutionarily conserved residues of S18 family genes in cancers

**Figure 8:**
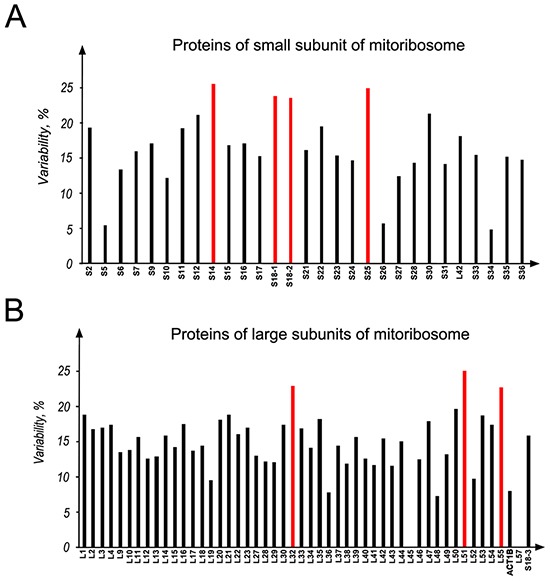
Mutational analysis of all the mitoribosomal whole proteome in different cancers **A.** MRPs of the small subunit; **B.** proteins of the large subunit of mitoribosome. Data were obtained from COSMIC database. The numbers of mutations, found for a mitochondrial ribosomal protein, were normalized to total length of that protein and the data were presented as percent of variability. Columns marked with red color are the proteins having more than 20% variability.

It is well known that cancer arises due to abnormalities in epigenetic and genetic mechanisms and also DNA mutations. Therefore, the mutational analysis of mitoribosomal proteins in biospecimens and different cancer cell lines was performed. All the 55S were analyzed, using the data of COSMIC database (Figure 8). The details of different kind of mutations in S18-1, -2 and -3 proteins are presented in Supplementary Table S4.

To find the proportion of variant to invariant of the mutations found in each protein, the mutation frequency was normalized to the total length of each protein. These data are presented as a percent of variability (Figure 8).

For the ease of clarification and comparison, mitoribosomal proteins were classified into two categories: (i) highly mutated proteins, if 20% or more variability was found and (ii) low mutated proteins, if less than 20% variability was found, according to the COSMIC database. In the S18 family, S18-1 and S18-2 are in the highly mutated category. Other highly mutated proteins of mitoribosome are MRPS14 and MRPS25 of the small subunit and MRPL32, MRPL51, and MRPL55 of the large subunit, as marked in red on Figure 8.

Noteworthy, the mutations at Gly132 (Supplementary Table S4) in the S18-2 sequence are often present in colon carcinoma samples: five out of six colon adenocarcinoma biopsies and one of ovarian carcinoma biopsies (deposited to COSMIC database) carried such mutations. Moreover, the ETA analysis (Figure 2 and Supplementary Figure S4) shows that this residue is highly conserved in the S18-2 protein sequence. Furthermore, Gly132 occupies the same position (24) in Ribosomal_S18 domains (Supplementary Figure S4) of all the S18 proteins, suggesting a fundamental role of this residue in the function of S18 proteins.

### Gly132 polymorphism in the S18-2 protein

Due to the fact that the Gly132 is often mutated in colorectal carcinoma (CRC), we have asked a question whether it is an important mutation, or does it represent polymorphism? To answer this question, DNA was amplified from both, normal and cancer tissues of CRC patients, using forward primers for wild-type and mutated *S18-2* gene.

**Figure 9:**
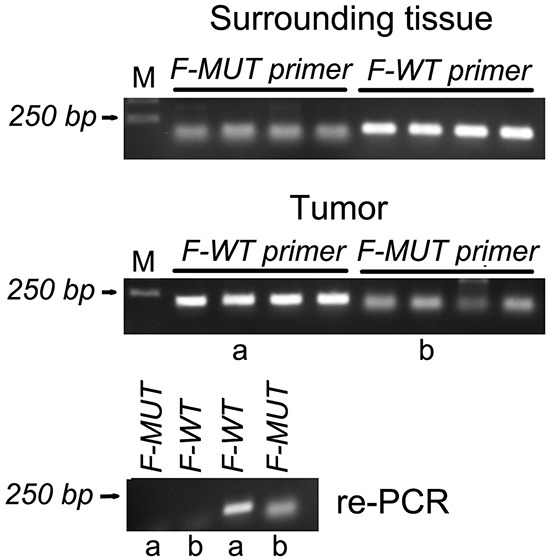
PCR amplification of the *S18-2* fragment in DNA isolated from normal tissues and tumor cells of four patients with colon carcinoma The top panel shows the PCR product of the *S18-2* gene from normal tissue, which were amplified using wild-type (F-WT) and mutant (F-MUT) primers. The middle panel is similar to the top panel, but DNA was isolated from tumor tissues. PCR products in lanes a and b were used for the second round of PCR. The bottom panel shows re-amplification of a and b PCR product with both, mutant and wild type primers.

When the mutated primer was used, amplification of *S18-2* was detected in 30 colon carcinoma samples and also in normal tissues of the same patients (examples are shown on Figure 9 and Supplementary Figure S7A). The same phenomenon was observed with the wild type primer. We have to mention, that the primers for recognition of the specific mutation were designed, as described in [14]. To validate the primers, two PCR products obtained from tumor tissue (see lanes **a** and **b** on Figure 9, the middle panel) were amplified again, using both, mutated and wild type primers. No products were obtained with alternative forward primers (see the bottom panel on Figure 9 and Supplementary Figure S7B).

For confirmation of primer specificity, the first PCR products (obtained with mutated or with wild type primers) from ten DNA samples (10 from tumor and also 10 from the matched normal tissues) were sequenced, and the presence of T-G substitution was detected. This mutation results in substitution of Gly to Cys at position 132.

The obtained data points on the presence of mutated DNA in both, normal and cancer tissues. Taking into consideration the Q-PCR and sequencing data, we can conclude that mutation is observed in approximately 0.1-1% of the cell DNA. It is lower than expected rate of germline and somatic mutation (heterozygous mutations should be observed in 50%, i.e. at least one allele should be mutated). We may speculate that genetic polymorphism (see [15]) is observed at Gly132.

## DISCUSSION

The S18 protein family consists of three homologs in Metazoa (S18-1–3) and one homolog in other cellular organisms. In the present study we explored the evolutionary history of S18 family in eukaryotic and in bacterial clades, and also analyzed the mutations of evolutionarily conserved residues in different cancer types.

The results show that the S18-1 is the most conserved metazoan homolog followed by S18-2 and S18-3. The Ribosomal_S18 domain is, by far, the most common domain found in S18 proteins of cellular organisms. Diversification and variation in domain architecture of bacterial S18 proteins points to possible acquisition of novel protein structure and functionality in bacterial S18; this should be investigated further in the light of cellular functions of Ribosomal_S18 domain containing proteins.

The phylogenetic analysis shows that ancient gene duplication followed by three rounds of the same event occurred in Metazoa during the evolution of eukaryotic S18. The most parsimonious reconciliation between gene tree and species tree shows that these duplications are metazoan-specific. This analysis also suggests that *S18-1* and *S18-2* resulted from recent gene duplication and *S18-3* emerged due to a relatively older gene duplication event. Same ancestral duplication is responsible for unexpected proximity between S18 homologs in Viridiplantae and Metazoan, compared with Metazoa and Fungi or with Viridiplantae and Protists in a strictly tree-like evolutionary model. Thus, ancient duplication is the most plausible cause of these unexpected events in the reconciliation of the S18 gene tree and the species tree.

It was shown recently that S18-1 and S18-2 proteins are present on a small subunit of mitoribosome, while S18-3 belongs to the large subunit [11]. The localization of S18-1 and S18-2 on the same subunit and S18-3 on the other subunit also indicates that *S18-3* appears in the result of earlier gene duplication. Its sibling remained a part of small subunit, where it underwent another gene duplication to branch into *S18-1* and *S18-2*. Furthermore, our study suggests that metazoan *S18-1* shares the most sequence similarity with *S18* of bacterial and of other eukaryotic clades. This is in agreement with a structural analysis showing that S18-1 occupies the position of its bacterial S18 homologs in the small ribosomal subunit [11].

Summarizing this part, we may conclude on the ancient gene duplication and the following three rounds of the same event in Metazoa, giving rise to three modern-day homologs of bacterial S18 in metazoan species.

Recent studies suggest that all three S18 homologs are zinc-binding proteins. Superposition of their structures revealed that they share a common zinc-binding core fold with the highly variable extensions. Noteworthy, it is quite rare when two protein chains form the zinc ion binding motif [11]. Probably, such interactions stabilize the structure of the rapidly evolving mitoribosomal proteins.

Taking into consideration the largely unknown functions of S18 proteins, the evolutionarily conserved amino acids of S18 family proteins were identified, using the published 3D structures [11]. As was discussed above, several residues are highly conserved in the S18-2 protein sequence. We wanted to understand whether these residues are mutated in tumors, hence, an analysis of the mutational status of all S18 proteins in various cancers and cancer cell lines was performed.

S18-2 was mutated at a higher rate compared to other S18 proteins. This is consistent with the hypothesis about relationship between gene duplication and the high mutation rate: gene duplication causes redundancy; one of the two gene duplicate attains the higher rate of mutation, leading to either gene loss (or pseudogenization) or acquisition of the new function (neofunctionalization or subfunctionalization), while the other gene duplicate remains conserved, due to selection pressure [16].

According to our analysis, the Gly132 residue of S18-2 was often mutated in CRC. We have to mention, that despite the long and successful identification of the main genes involved in development of CRC, between 20% and 50% of cases fail to show mutations in these genes by currently available technologies. There is an opinion that heredity is responsible for approximately one-third of the susceptibility to CRC [17]. The causative germ-line mutations account for less than 6% of all CRC cases [18]. Obviously, there should be other genes that are responsible for risk of CRC acquisition, when mutated. Few such genes have been detected recently, for example, *MYH* and *EPCAM*. Importantly, several studies were conducted, using the relatively large, unselected series of CRC patients. Patterns of polymorphisms in candidate and anonymous genes spread throughout the genome were analyzed, based on International HapMap Project data [19].

We found the Gly132Cys mutation in all 30 CRC samples and in the corresponding surrounding normal tissues. These data were supported by direct DNA sequencing. However, the frequency of mutation was approximately 1% or less, as was shown by q-PCR, which suggests the genetic polymorphism of the Gly132 residue of S18-2. The further studies are required to show the functionality of this polymorphism. We have to mention, that we compared the *S18-2* DNA sequence with the reference gene at NCBI home page, so germline or somatic mutation could be excluded here.

As mentioned above, the overexpressed of S18-2 protein induced chromosomal instability in primary cells, resulting in their transformation [8–10]. Recently, we have found that the S18-2 protein was expressed at the higher levels in endometrial cancers, compared to hyperplasia and normal tissues [20]. Moreover, the S18-2 protein expressed at high levels induced epithelial-mesenchymal transition of the endometrial carcinoma cells *in vitro*. The role of S18-2 protein in CRC development should be further elucidated.

## CONCLUSIONS

Inferring evolutionary history of a gene is the initial step to predict the functional and structural properties of the encoded protein. Therefore, three *Homo sapiens* homologous S18 proteins were traced in the tree of life. In result of analysis, using bioinformatic tools, we concluded on the ancient gene duplication and the following three rounds of the same event in Metazoa, giving rise to three modern-day S18 proteins in metazoan species.

Evolutionary trace analysis showed that the S18-2 protein is mutated at the higher rate in tumors, compared with S18-1 and S18-3. The one of the most conserved residues in the S18-2 protein sequence, Gly132, is often mutated in CRC. We found the genetic polymorphism Gly132Cys in clinical CRC samples and the surrounding normal tissues. Further studies are required to assess the functionality of this polymorphism and a possible use as a CRC biomarker.

## MATERIALS AND METHODS

### S18 homolog identification and species tree construction

The Basic Local Alignment Search Tool (BLAST) search [21] was performed, using the *Homo sapiens* S18-1, -2 and -3 proteins as seed sequences to get orthologs in all selected species, using NCBI NR database (protein IDs are provided in Supplementary Table S1 and S2). Proteins with partial sequences were rejected. Each bacterial S18 protein used in this study is named by S18 and taxonomic class, separated by underscore. For example, S18_Alphaproteobacteria means the S18 homolog in Alphaproteobacteria. Similarly, each eukaryotic S18 protein used in this study is named by S18 and the initials of complete species name separated by underscore; for example, S18-1_Hom-sap represents S18-1 protein in Homo sapiens, etc. Eukayotes were identified and used in this study to infer and differentiate the evolutionary and duplication patterns in Metazoan S18 proteins from its non-metazoan homologs.

### Domain architecture retrieval

Domain architecture of all S18 proteins was extracted, using the Batch search tool available at NCBI Conserved Domain Database (CDD) [22] and the Superfamily web tool available at HMM Library and Genome Assignment Server [23].

### Evolutionary trace analysis (ETA)

The ETA is a phylogenomic method used to identify evolutionarily conserved residues in protein sequences. It is a theoretical approach for the validation of experimental mutations. ETA identifies the globally conserved residues of proteins, and pinpoints class-specific residues that are important in defining the unique properties of proteins of interest [24, 25]. The ETA was used to extract evolutionarily conserved residues of S18 homologs. *Homo sapiens* S18-1, -2, and -3 protein sequences were provided to the ETA server available at http://www.mammoth.bcm.tmc.edu/ to find all evolutionarily conserved residues in proteins. The BLAST search was used to obtain homologous sequences with sequence identity >50% and E-value <1 · e^−3^. Sequences were aligned, using the MUSCLE software at the ETA server. S18-1, -2 and -3 sequences of *Sus scrofa* was used as query sequence and its crystallographic structure (PDB IDs: 5AJ3R for S18-1, 5AJ3p of S18-2 and 4V1Ax for S18-3) were used for structural mapping. For structural analysis, the Pymol tool was used that is employed at the ETA webserver [26]. The ETA was performed also for *Homo sapiens* Ribosomal_S18 domain of S18 protein family. For this purpose, the multiple sequence alignment (MSA) generated for phylogenetic analysis was provided to the Hidden Markov Models (HMM) logos server [13].

### Phylogenetic analysis

Phylogenetic analyses were performed using a pipeline, consisting of MEGA6 [27], TimeTree [28], DLRS in JPrIMe [29, 30] and Visual Markov Chain Monte Carlo (VMCMC) (not published yet). Briefly, S18 protein sequences in bacteria and in eukaryotes were aligned, using ClustalW tool employed in MEGA software with affine gap penalties of 5 for gap opening and 1 for gap extension [31]. Bacterial species tree (Supplementary Figure S1) was retrieved from the TimeTree, using the expert suggestions for probable divergence times between species, which is required for Markov Chain Monte Carlo (MCMC) analysis in JPrIMe-DLRS. The eukaryotic species tree (Supplementary Figure S2) was constructed by combination of the tree topology and divergence time information for Protists, Porifera, Viridiplantae and Fungi described in [32]. Noteworthy, a different lineage for Protists is used in [32], than what is inferred from expert suggestion from the TimeTree [28], where Protists have diverged more recently from Viridiplantae rather than with the last common ancestor of Viridiplantae and Opisthokonta. The estimates are given by the TimeTree for Metazoa and Placozoa. One million MCMC iterations were run on the JPrIME-DLRS with default parameter settings for bacterial S18 proteins and five million MCMC iterations for eukaryotic S18 proteins. The initial 25% samples in both MCMC runs for bacterial and eukaryotic S18 proteins were removed as burning; convergence was checked then, using diagnostics implemented in the VMCMC tool. The Maximum a Posteriori (MAP) tree and the consensus tree were determined by analysis of the output tree distribution, using the VMCMC tool. Finally, the state with Maximum Likelihood (ML) of MCMC chain was selected from the JPrIMe-DLRS run. The cladogram, representing all of the phylogenetic trees was drawn, using Archaeopteryx application in Forester library [33].

### Most parsimonious reconciliation analysis

For deducing the evolutionary history and patterns of gene duplications in eukaryotic S18 family of proteins, the most parsimonious reconciliation (MPR) between eukaryotic species tree and consensus gene tree was computed, using the NOTUNG [34] with default settings. The MPR was introduced by Goodman *et al.* [35], who pioneered the gene-tree – species-tree comparisons. The MPR represents the mapping between each gene-tree vertex to either a species tree vertex (speciation) or to a species tree edge (gene duplication) in a parsimonious way (minimizing the number of duplications). The MPR is preferred to other reconciliations, because in most cases the MPR represents the true reconciliation between gene tree and the species tree [36, 37].

### Mutational analysis

To study the mutations carried by all proteins of small and large subunits of mitoribosome in different cancer types, data at web-based Catalogue of Somatic Mutations in Cancer (COSMIC) (http://cancer.sanger.ac.uk) [38] were analyzed. Samples from benign neoplasm, *in situ* and invasive tumors, recurrences, metastases, and cancer cell lines are all included in COSMIC database. Briefly, the database gathers information from two sources. Firstly, mutations in notorious cancer genes are collected from the literature. The genes are identified by their presence in Cancer Gene Census. Secondly, the data are included in the database after whole-genome sequencing of cancer samples that is performed by Cancer Genome Project [38]. The number of mutations reported for all the proteins of large and small subunit of mitochondrial ribosome was extracted from COSMIC database. Briefly, the mutation for each mitoribosome was individually searched in COSMIC database, using the gene symbol for each protein. The percent variability of each protein is presented. The number of mutations reported in the COSMIC database was normalized to the total length (number of amino acids) of protein (as denominator).

### DNA extraction, primer design, PCR amplification and DNA sequencing

Tumor samples were obtained at National Cancer Institute of Ministry of Health of Ukraine, Kyiv. Diagnosis was made by an experienced pathologist, based on hematoxylin/eosin staining of tissues sections (see Supplementary Table S5). All patients have given the written informed consent. The Ethics Committee of the R.E. Kavetsky IEPOR of NASU (Kyiv, Ukraine) approved the present study.

DNA was isolated from the thirty surgically removed tumors (colon adenocarcinomas at the stage I-IV) and the surrounding normal tissue, using QIAamp DNA FFPE Tissue Kit (QIAGEN, Hilden, Germany), according to the manufacturer protocol.

Two different sets of primers (wild type and mutated primers) were designed for amplification of *S18-2* gene. The first nucleotide of Gly132 codon was positioned as the last nucleotide at 3′ end of both forward primers. Both primers had same sequence except the two nucleotides at 3′ (indicated in bold) in mutant primer; the reverse primers were both of wild type. The primers sequences were, wild_F: 5′-TTGTCTGCGCCCACACGG-3′; mutant_F: 5′- TTGTCTGCGCCCACA**T**G**T**-3′, to monitor G-T substitution as the most frequent mutation in the database. Other types of mutation were not checked. Reverse primer was 5′-AGGAGCCACTGAACAAATACCT-3′. For one amplification reaction 0.5μg of DNA, 3μM of each primer and 1X Maxima SYBR green PCR master mix (Fermentas, Vilnius, Lithuania) was used. Applied Biosystem 7500 thermocycler machine (Applied Biosystems, Foster City, CA, USA) was used for amplification with following cycling program: 20°C for 25 min, 95°C for 10 min, and 40 cycles at 95°C for 30 s, 60°C for 30 s, and 72°C for 30 s. The PCR products were separated on 0.8% agarose gel for visualization.

The PCR products were extracted using GeneJET PCR Purification Kit (Fermentas), following supplier protocol. This PCR product was used for primer validation as well as for sequencing. To validate wild type primer, the product of PCR that were previously amplified with mutant primer was re-amplified with wild type primer (Supplementary Figure S7B, the top panel). Correspondingly, for validation of mutant primer, the product of PCR amplified with wild type primer was re-amplified with mutant primer (Supplementary Figure S7B, the bottom panel). As positive control, PCR products were also re-amplified with the same set of primers that were used for the first PCR (Supplementary Figure S7B).

Only the reverse primer was used to sequence the first round PCR products. Sequencing was performed, using the BigDyeTM terminators v.3.1 and 1.1 (Applied Biosystems) and ABI 3730 DNA analyzer at gene facility of Karolinska Institutet. Chromas software was used to read the chromatograms. As only reverse primer was used for sequencing, the reverse complement sequence was generated in Fasta format. Finally, sequences were subjected to NCBI BLAST for identification of mutations.

## SUPPLEMENTARY FIGURES AND TABLES








