# Supplementary material for: S18 family of mitochondrial ribosomal proteins: evolutionary history and Gly132 polymorphism in colon carcinoma
Source: Oncotarget. 2016 Jul 30;7(34):55649–62. doi: 10.18632/oncotarget.10957 (PMC5342443; doi:10.18632/oncotarget.10957)
Supplement: Supplementary file 1 [file oncotarget-07-55649-s001.pdf]

S18 family of mitochondrial ribosomal proteins: evolutionary history and Gly132 polymorphism in colon carcinoma

SUPPLEMENTARY FIGURES AND TABLES

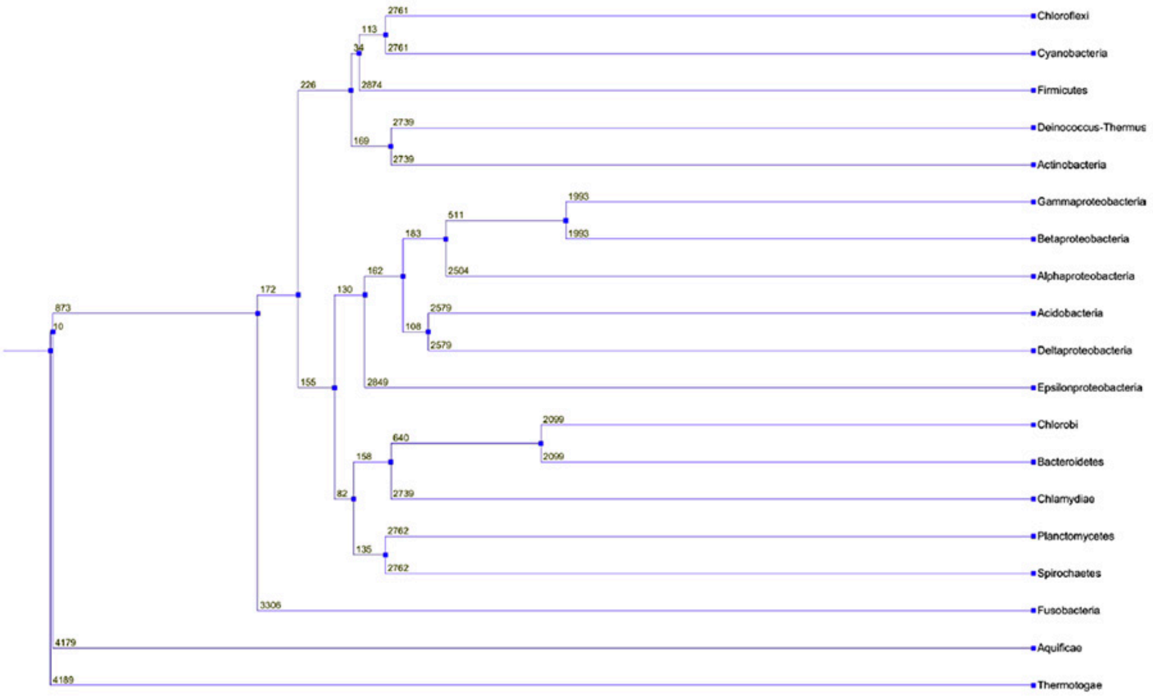

Supplementary Figure S1: Bacterial species tree as a cladogram, consisting of bacterial species and divergence time on branches. Following is the Newick string of the bacterial taxonomic tree we have employed in this study for JPrIMe-DLRS. It was determined, using NCBI Taxonomy database for tree taxonomy and from TimeTree to infer divergence times. (((((((Chloroflexi: 2761, Cyanobacteria: 2761): 113, Firmicutes: 2874): 34, (Deinococcus Thermus: 2739, Actinobacteria: 2739): 169): 226, (((((Gamma proteobacteria: 1993, Beta proteobacteria: 1993): 511, Alpha proteobacteria: 2504): 183, (Acidobacteria: 2579, Delta proteobacteria: 2579): 108): 162, Epsilon proteobacteria: 2849): 130, (((Chlorobi: 2099, Bacteroidetes: 2099): 640, Chlamydiae: 2739): 158, (Planctomycetes: 2762, Spirochaetes: 2762): 135): 82): 155): 172, Fusobacteria: 3306): 873, Aquificae: 4179): 10, Thermotogae: 4189): 1000.

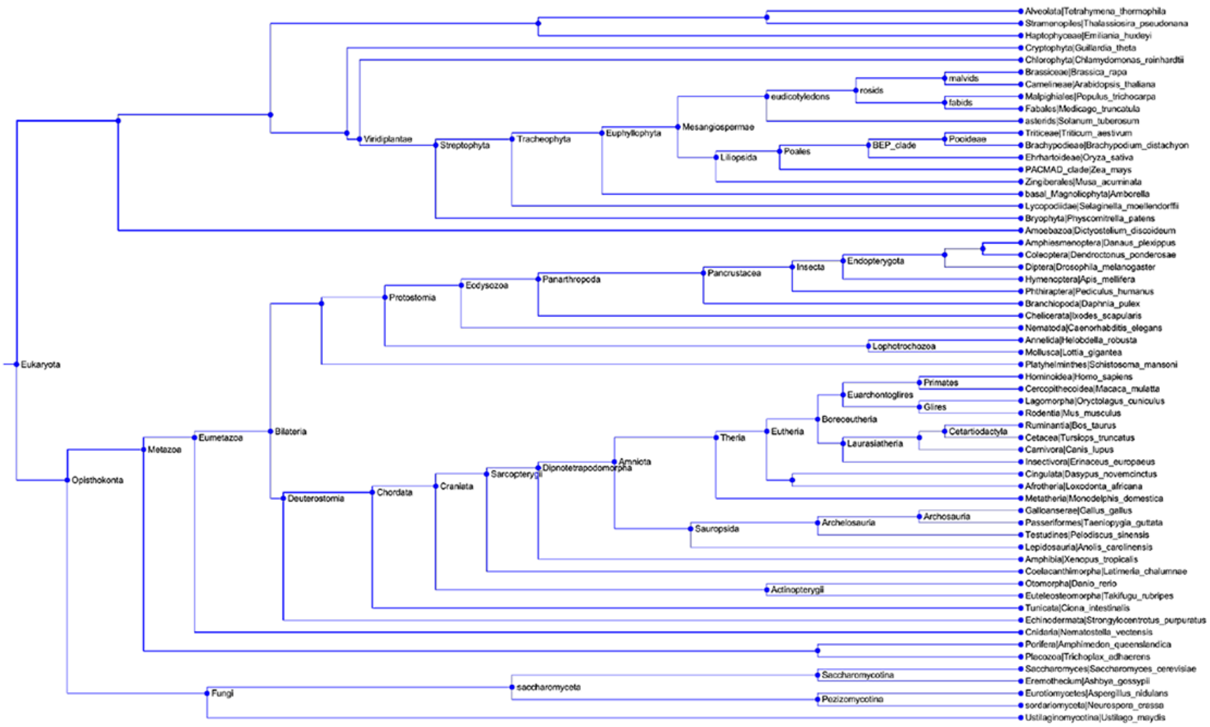

Supplementary Figure S2: Eukaryotic species tree as a cladogram, consisting of eukaryotic species and taxonomic clade nameson branches. The leaf names indicate the taxonomic class of leaf and the species name, in which S18 proteins were found. Following is the Newick string of the eukaryotic species tree we have employed in this study for JPrIMe-DLRS. The topology for most of the species in this tree have been taken from Parfrey et. al and the rest from TimeTree.org and NCBI Taxonomy database. ((Dictyostelium discoideum: 1773.15413620058, (((Tetrahymena thermophila: 1469.642457632497, Thalassiosirapseudonana: 1469.642457632497): 76.2460363750481, Emiliania huxleyi: 1545.8884940075475): 80.15423791873536, (Guillardia theta: 1338.2692704438693, (Chlamydomonas reinhardtii: 936.3041909585723, (Selaginella moellendorfii: 593.4, (Physcomitrella patens: 433.07, (Amborella: 150.1, ((Musa acuminata: 116.0, (Oryza sativa: 34.1, (Zea mays: 31.6, (Triticum aestivum: 24.0, Brachypodium-distachyon: 24.0): 7.6) : 2.5): 81.9): 31.8, (Solanum-tuberosum: 125.0, ((Populus trichocarpa: 98.0, Medicago truncatula: 98.0): 11.0, (Brassica rapa: 16.4, Arabidopsis thaliana: 16.4): 92.6): 16.0): 22.8): 2.3): 282.97): 160.33): 342.9041909585723): 401.965079485297): 287.77346148241213): 147.11140427429223): 7.937535486708384, ((Ustilago maydis: 771.7234420252413, ((Aspergillus nidulans: 402.0, Neurospora crassa: 402.0): 271.0, (Saccharomyces cerevisiae: 311.0, Ashbya gossypii: 311.0): 362.0): 98.7234420252413): 523.9881694689175, (Amphimedon queenslandica: 1237.0, (Trichoplax adhaerens: 1147.0, (Nematostella vectensis: 784.6400568398692, ((Schistosoma mansoni: 632.2351224058409, ((Ciona intestinalis: 600.0, Strongylocentrotus purpuratus: 600.0): 29.23512240584145, ((Helobdella robusta: 545.0, Lottia-gigantea: 545.0): 65.0, (Caenorhabditis elegans: 555.1623337181048, (Ixodes scapularis: 535.0, (Daphnia pulex: 470.0, (Pediculus humanus: 355.0, (Apis mellifera: 310.7051708776171, (Drosophila melanogaster: 300.0, (Danaus plexippus: 280.0, Dendroctonus ponderosae: 280.0): 20.0): 10.7051708776171): 44.2948291223829): 115.0): 65.0): 20.1623337181048): 54.8376662818952): 19.23512240584145): 2.99999999999945): 59.80443141904982, ((Danio rerio: 307.0, Takifugu-rubripes: 307.0): 147.6, (Latimeria chalumnae: 430.0, (Xenopus tropicalis: 361.2, ((Anolis carolinensis: 274.9, (Pelodiscus sinensis: 230.7, (Gallus gallus: 95.2, Taeniopygia guttata: 95.2): 135.5): 44.2): 49.6, (Monodelphis domestica: 176.1, ((Dasypus novemcinctus: 103.3, Loxodonta africana: 103.3): 1.4, ((Erinaceus europaeus: 87.2, (Canis lupus: 84.6, (Bos taurus: 59.1, Tursiops truncatus: 59.1): 25.5): 2.6): 10.2, ((Oryctolagus cuniculus: 86.4, Mus musculus: 86.4): 4.6, (Homo sapiens: 29.6, Macaca mulatta: 29.6): 61.4): 6.4): 7.3): 71.4): 148.4): 36.7): 68.8): 24.6): 237.43955382489128): 92.60050301497768): 362.3599431601308): 90.0): 58.7116114941582): 485.380060193123): 1000.0.

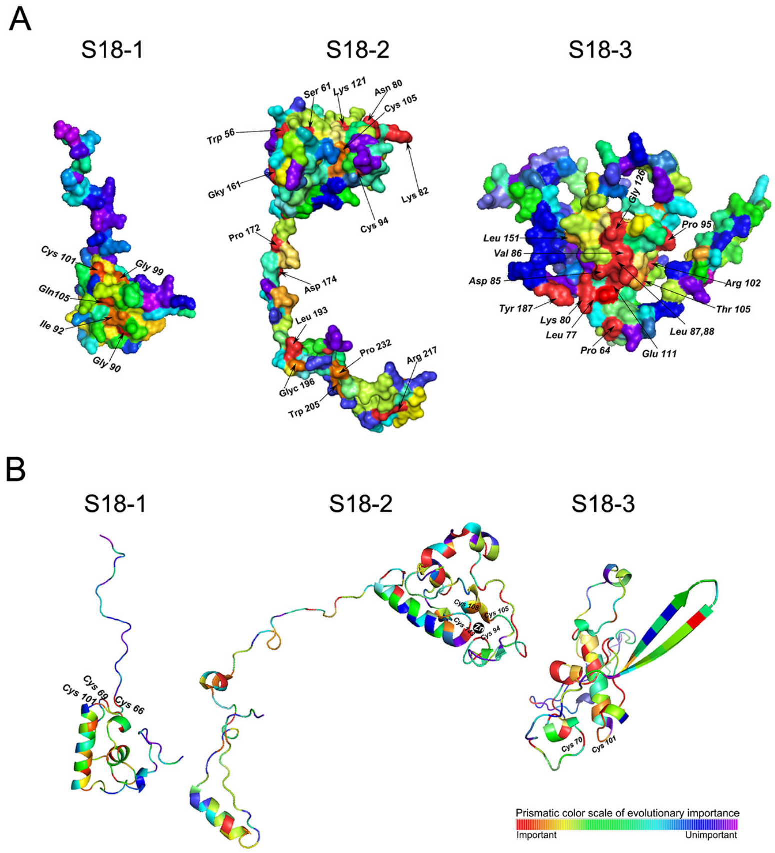

Supplementary Figure S3: ETA structural analysis of S18 proteins. Crystallographic structures were extracted from Greber B.J *et al*. 2015. **A.** Surface view of the structure of S18 family of proteins. Structures are colored as heat diagrams with red residues as evolutionarily conserved traces. **B.** Ribbon cartoons of the same structures, labeled amino acids are Cys residues of S18 proteins that are involved in the Zinc finger motif. The structures were developed using Pymol software.

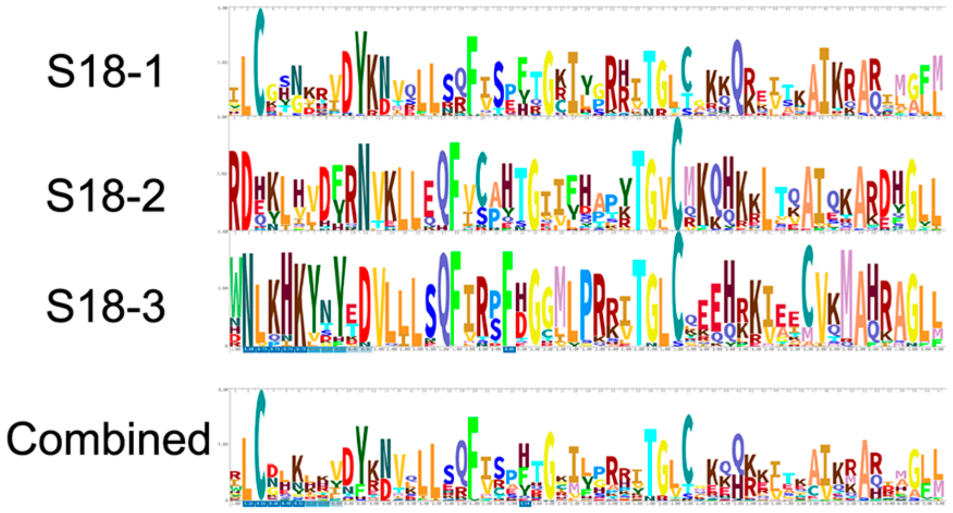

Supplementary Figure S4: HMM logos for Ribosomal S18 domain of S18 proteins. The Gly at the position (24) is the evolutionary conserved residue of combined HMM logos for the ribosomal S18 family of proteins.

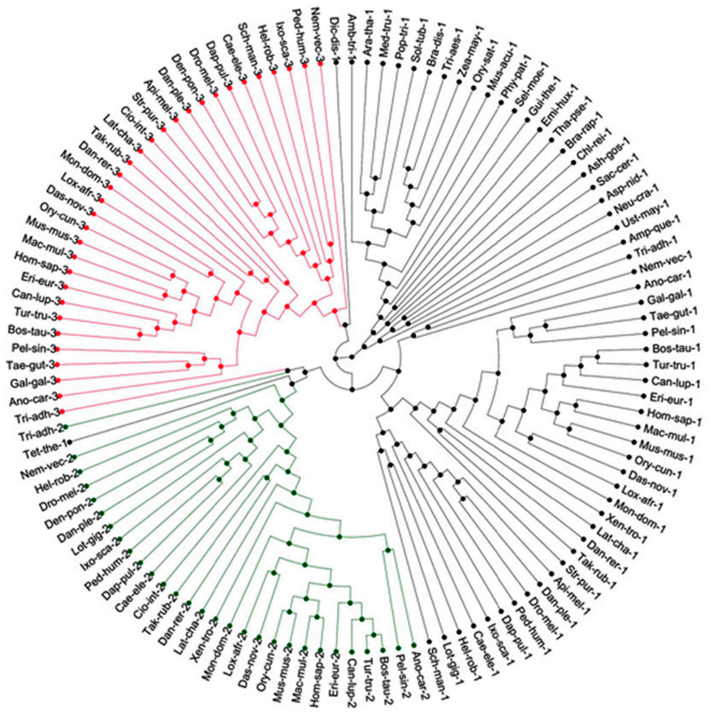

Supplementary Figure S5: Maximum-A-Posteriori tree and tree at state with maximum likelihood. A circular cladogram representation of the Maximum-A-Posteriori (MAP) tree, which is also the tree at the state with Maximum Likelihood in the posterior. We have color-coded this tree according to the scheme: Black → MRSP18-1, Green → MRSP18-2 and Red → MRSP18-3 to show that all proteins are grouping with their orthologs and that MRSP18-1 is more similar to MRSP18-2 than MRSP18-3.

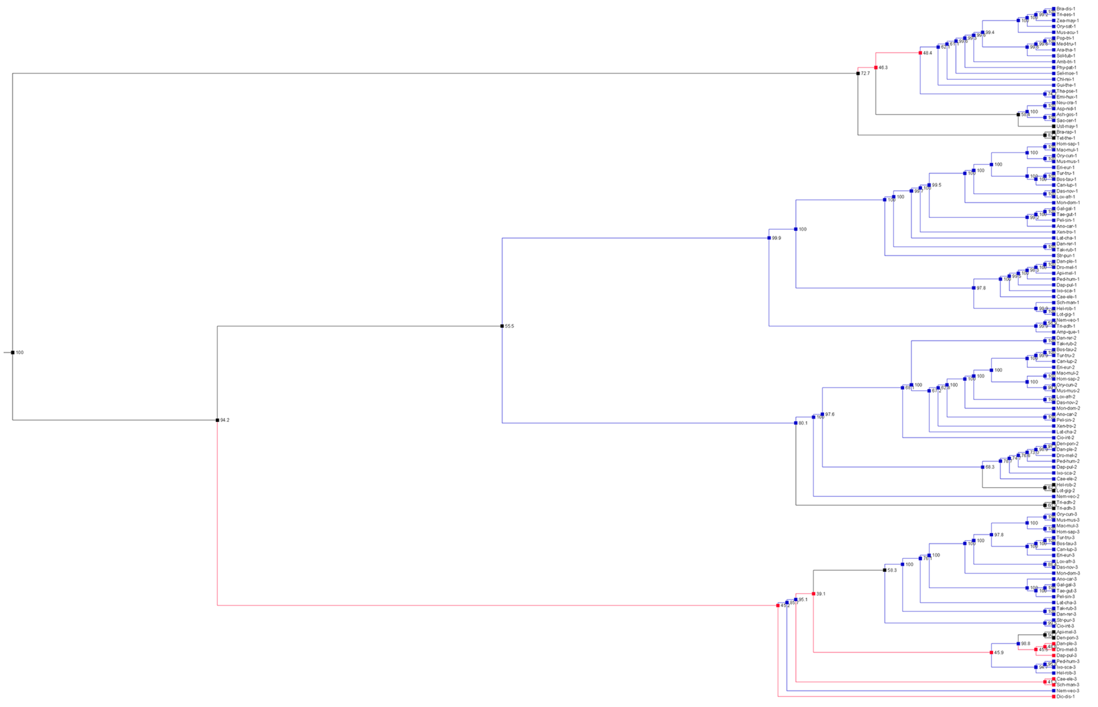

Supplementary Figure S6: S18 eukaryotic consensus tree. Except for a few splits close to root of species tree, the majority of splits are highly supported in tree posterior with posterior frequency greater than 80%.

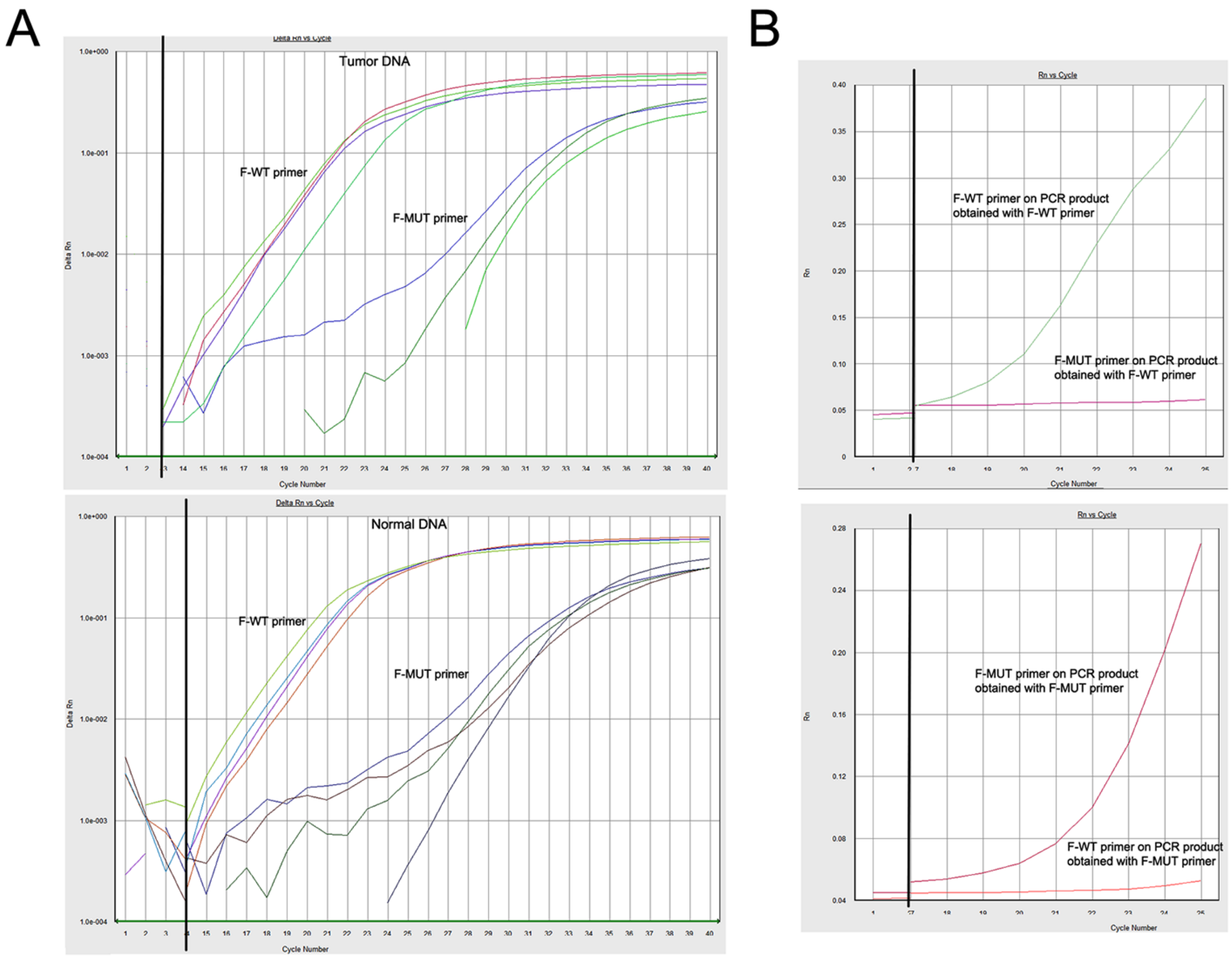

Supplementary Figure S7: Q-PCR on tumor samples of colon adenocarcinoma and corresponding normal tissue. **A.** S18-2 genomic DNA was amplified with both, mutant (F-MUT) and wild type (F-WT) forward primers. PCR amplification on few samples of tumor DNA is shown on the top panel; amplification on DNA from corresponding normal tissue is shown on the bottom panel. **B.** Re-amplification of two PCR products obtained by amplification of tumor DNA. The top panel – the first PCR product was obtained the wild type primer; the bottom panel – the first PCR product was amplified with the mutant primer.

Supplementary Table S1: Bacterial S18 proteins. The table displays the taxonomic diversity and clades of bacteria that possess *S18* gene.

No	GI	Accession ID	Representative Species	Bacterial Clade
1	445990809	WP_000068664	*Firmicutes*	Firmicutes
2	488794038	WP_002706444	*Treponema saccharophilum*	Spirochaetes
3	493797913	WP_006745891	*Thioalkalivibrio*	Gammaproteobacteria
4	495334257	WP_008058994	*Methyloversatilis*	Betaproteobacteria
5	501575308	WP_012579695	*Thermosipho africanus*	Thermotogae
6	501591037	WP_012594993	*Cyanothece*	Cyanobacteria
7	511068541	WP_016310128	*Enterorhabdus*	Actinobacteria
8	518820616	WP_019976570	*Empedobacter*	Bacteroidetes
9	548240343	WP_022458510	*Fusobacterium sp. CAG:439*	Fusobacteria
10	551219330	WP_022847268	*Desulfurobacterium sp. TC5-1*	Aquificae
11	652407344	WP_026803084	*Arcobacter*	Epsilonproteobacteria
12	653296164	WP_027481122	*Deinococcus pimensis*	Deinococcus-Thermus
13	658545513	WP_029738249	*Chloroflexi bacterium JGI 0002000-F10*	Chloroflexi
14	737616672	WP_035587240	*Hippea jasoniae*	Deltaproteobacteria
15	737740036	WP_035708741	*Haematobacter*	Alphaproteobacteria
16	739339800	WP_037202082	*Rhodopirellula*	Planctomycetes
17	740204708	WP_038046653	*Thermoanaerobaculum aquaticum*	Acidobacteria
18	752832630	WP_041468932	*Chloroherpeton thalassium*	Chlorobi
19	754883876	WP_042243026	*Neochlamydia*	Chlamydiae

GI – the gene identification number; Accession ID – protein accession number

Supplementary Table S2: Eukaryotic S18 proteins. The table displays the taxonomic diversity and clades that possess *S18* genes.

See Supplementary File 1

Supplementary Table S3: The evolutional trace analysis of human proteins S18-1, S18-2 and S18-3, based on the 3D structure

S18-1	S18-2	S18-3
Cys65	Tyr58	Cys70
Cys68	Pro72	Pro71
Lys75	Arg78	Lys80
Lys76	Arg79	Asp85
Leu79	Asn80	Val86
Leu80	Gly83	Leu87
Gln82	Pro86	Leu88
Phe83	Thr90	Leu89
Ser85	Arg91	Gln91
Thr88	Cys94	Phe92
Gly89	Leu122	Arg94
Tyr92	Leu123	Gly97
His95	Gln125	Leu100
Ile96	Phe126	Pro101
Thr97	Thr131	Arg102
Gly98	Gly132	Thr105
Leu99	Thr140	Gly106
Cys100	Gly141	Leu107
Gln104	Cys143	Cys108
Ile111	Gln146	Glu111
Met120	Lys148	Val119
Lys125	Leu150	Met121
Asp142	Ala157	Ala122
Pro143	Gly161	Ala124
	Leu162	Leu126
	Pro168	Pro129
	Asp176	Pro133
		Leu147

Supplementary Table S4: Specific mutations in S18-1, S18-2, and S18-3 proteins in the different types of cancer and also in cancer cell lines. The data of the COSMIC database was analyzed. The G132 mutation in S18-2, which is widely present in colon carcinomas, is marked in bold.

See Supplementary File 2

Table S5. The samples of colon carcinoma, studied in the present work. All 30 samples were studied. Ten samples for those the PCR products, obtained with forward wild type and mutated primers, were sequenced are indicated in bold.

See Supplementary File 3
