# Supplementary material for: S18 family of mitochondrial ribosomal proteins: evolutionary history and Gly132 polymorphism in colon carcinoma
Source: Oncotarget. 2016 Jul 30;7(34):55649–62. doi: 10.18632/oncotarget.10957 (PMC5342443; doi:10.18632/oncotarget.10957)
Supplement: Supplementary file 2 [file oncotarget-07-55649-s002.docx]

**Table S2. Eukaryotic S18 proteins.** The table displays the taxonomic diversity and clades that possess *S18* genes.

| **No** | **Species Name** | **Taxonomic Clade** | **Protein ID** | | |
| --- | --- | --- | --- | --- | --- |
|  |  |  | **S18-1** | **S18-2** | **S18-3** |
| 1 | *Tetrahymena thermophila* | Alveolata | XP_001017206 | - | - |
| 2 | *Thalassiosira pseudonana* | Stramenopiles | YP_874526 | - | - |
| 3 | *Emiliania huxleyi* | Haptophyceae | YP_277328 | - | - |
| 4 | *Guillardia theta* | Cryptophyta | NP_050745 | - | - |
| 5 | *Chlamydomonas reinhardtii* | Chlorophyta | NP_958392 | - | - |
| 6 | *Brassica rapa* | Brassiceae | XP_009118457 | - | - |
| 7 | *Arabidopsis thaliana* | Camelineae | NP_051081 | - | - |
| 8 | *Populus trichocarpa* | Malpighiales | YP_001109524 | - | - |
| 9 | *Medicago truncatula* | Fabales | YP_001381706 | - | - |
| 10 | *Solanum tuberosum* | Asterids | YP_635661 | - | - |
| 11 | *Triticum aestivum* | Triticeae | NP_114280 | - | - |
| 12 | *Brachypodium distachyon* | Brachypodieae | YP_002000507 | - | - |
| 13 | *Zea mays* | PACMAD clade | NP_043046 | - | - |
| 14 | *Oryza sativa* | Ehrhartoideae | NP_039408 | - | - |
| 15 | *Musa acuminata* | Zingiberales | ABU85446 | - | - |
| 16 | *Amborella* | Basal Magnoliophyta | NP_904121 | - | - |
| 17 | *Physcomitrella patens* | Bryophyta | NP_904178 | - | - |
| 18 | *Selaginella moellendorﬃi* | Lycopodiidae | ADH10399 | - | - |
| 19 | *Dictyostelium discoideum* | Amoebazoa | X_ 646259 | - | - |
| 20 | *Danaus plexippus* | Amphiesmenoptera | EHJ68500 | EHJ70696 | EHJ76625 |
| 21 | *Dendroctonus ponderosae* | Coleoptera | - | ENN76200 | AEE62851 |
| 22 | *Drosophila melanogaster* | Diptera | NP_524593 | NP_523606 | NP_731252 |
| 23 | *Apis mellifera* | Hymenoptera | XP_003249593 | - | XP_623794 |
| 24 | *Pediculus humanus* | Phthiraptera | XP_002432087 | XP_002426373 | XP_002428056 |
| 25 | *Daphnia pulex* | Branchiopoda | EFX77265 | EFX88791 | EFX71554 |
| 26 | *Ixodes scapularis* | Chelicerata | XP_002410487 | XP_002414261 | XP_002402126 |
| 27 | *Caenorhabditis elegans* | Nematoda | NP_495374 | NP_495800 | NP_498835 |
| 28 | *Helobdella robusta* | Annelida | XP_009022051 | XP_009031159 | XP_009021887 |
| 29 | *Lottia gigantea* | Mollusca | XP_009052377 | XP_009044501 | - |
| 30 | *Ciona intestinalis* | Tunicata | - | XP_002129978 | XP_002128137 |
| 31 | *Strongylocentrotus purpuratus* | Echinodermata | XP_792269 | - | XP_011680394 |
| 32 | *Schistosoma mansoni* | Platyhelminthes | CCD79786 | - | CCD74777 |
| 33 | *Homo sapiens* | Hominoidea | NP_057151 | NP_054765 | NP_060605 |
| 34 | *Macaca mulatta* | Cercopithecoidea | NP_001253055 | NP_001098635 | NP_001244564 |
| 35 | *Oryctolagus cuniculus* | Lagomorpha | XP_008265846 | XP_002714378 | XP_002714485 |
| 36 | *Mus musculus* | Rodentia | NP_081102 | NP_080154 | NP_081044 |
| 37 | *Bos taurus* | Ruminantia | NP_001039973 | NP_001033613 | NP_001094699 |
| 38 | *Tursiops truncatus* | Cetacea | XP_004328001 | XP_004323727 | XP_004315064 |
| 39 | *Canis lupus* | Carnivora | XP_535635 | XP_532057 | XP_532152 |
| 40 | *Erinaceus europaeus* | Insectivora | XP_007537557 | XP_007532392 | XP_007530471 |
| 41 | *Dasypus novemcinctus* | Cingulata | XP_004472735 | XP_012377436 | XP_004473127 |
| 42 | *Loxodonta africana* | Afrotheria | XP_003414155 | XP_003422520 | XP_003404215 |
| 43 | *Monodelphis domestica* | Metatheria | XP_007496015 | XP_001375521 | XP_001366285 |
| 44 | *Gallus gallus* | Galloanserae | NP_001239240 | - | NP_001264552 |
| 45 | *Taeniopygia guttata* | Passeriformes | XP_012428738 | - | NP_001232301 |
| 46 | *Pelodiscus sinensis* | Testudines | XP_006110975 | XP_006111823 | XP_006131409 |
| 47 | *Anolis carolinensis* | Lepidosauria | XP_008109215 | XP_003217994 | XP_003229291 |
| 48 | *Xenopus tropicalis* | Amphibia | NP_001017092 | XP_002939509 | - |
| 49 | *Latimeria chalumnae* | Coelacanthimorpha | XP_005999428 | XP_006011869 | XP_005999413 |
| 50 | *Danio rerio* | Otomorpha | NP_001038655 | NP_001017759 | NP_001025285 |
| 51 | *Takifugu rubripes* | Euteleosteomorpha | XP_003965509 | XP_011607659 | XP_003971609 |
| 52 | *Nematostella vectensis* | Cnidaria | XP_001639669 | XP_001636328 | XP_001628605 |
| 53 | *Amphimedon queenslandica* | Porifera | XP_011402993 | - | - |
| 54 | *Trichoplax adhaerens* | Placozoa | XP_002114553 | XP_002116029 | XP_002110195 |
| 55 | *Saccharomyces cerevisiae* | Saccharomyces | AJV34138 | - | - |
| 56 | *Ashbya gossypii* | Eremothecium | NP_983979 | - | - |
| 57 | *Aspergillus nidulans* | Eurotiomycetes | XP_680718 | - | - |
| 58 | *Neurospora crassa* | sordariomyceta | XP_961619 | - | - |
| 59 | *Ustilago maydis* | Ustilaginomycotina | XP_011388078 | - | - |
