# Supplementary material for: S18 family of mitochondrial ribosomal proteins: evolutionary history and Gly132 polymorphism in colon carcinoma
Source: Oncotarget. 2016 Jul 30;7(34):55649–62. doi: 10.18632/oncotarget.10957 (PMC5342443; doi:10.18632/oncotarget.10957)
Supplement: Supplementary file 3 [file oncotarget-07-55649-s003.docx]

**Table S4.** Speciﬁc mutations in S18-1, S18-2, and S18-3 proteins in the different types of cancer and also in cancer cell lines. The data of the COSMIC database was analyzed. The G132 mutation in S18-2, which is widely present in colon carcinomas, is marked in bold.

| **Type of mutation** | **Sample Name** | **Mutation site** | **Cancer type** |
| --- | --- | --- | --- |
| **S18-1 protein** | | | |
| ***Tumors*** | | | |
| Synonymous substitution | TCGA-D8-A1JS-01 | A2A | Breast carcinoma |
| Synonymous substitution | ICGC MB81 | G93G | Brain tumor |
| Synonymous substitution | TCGA-EE-A2MI-06 | K14K | Malignant melanoma |
| Missense substitution | TCGA-A8-A09Z-01 | A114T | Breast carcinoma |
| Missense substitution | TCGA-D1-A17B-01 | R141Q | Endometrial carcinoma |
| Missense substitution | TCGA-CZ-5461-01 | Q78K | Renal clear cell carcinoma |
| Missense substitution | TCGA-BP-4988-01 | P121S | Renal clear cell carcinoma |
| Missense substitution | TCGA-A6-5661-01 | K14E | Colon carcinoma |
| Missense substitution | TCGA-AA-3715-01 | F119V | Colon carcinoma |
| Missense substitution | HCC19 | G39S | Liver carcinoma |
| Missense substitution | HCC19T | G39S | Liver carcinoma |
| Missense substitution | RK212 C01 | I138S | Liver carcinoma |
| Missense substitution | TCGA-D3-A2JN-06 | P51L | Malignant melanoma |
| Missense substitution | TCGA-BR-6452-01 | C65R | Stomach adenocarcinoma |
| Missense substitution | TCGA-55-7815-01 | A22T | Lung carcinoma |
| Missense substitution | TCGA-EE-A3J7-06 | L16S | Malignant melanoma |
| Missense substitution | TCGA-GV-A3QI-01 | I111N | Urinary tract carcinoma |
| Nonsense substitution | Z138 | C8* | Mantle cell lymphoma |
| Nonsense substitution | TCGA-AG-A002-01 | E106* | Colon carcinoma |
| Deletion frame shift | TCGA-D8-A1Y1-01 | K15delK | Breast carcinoma |
|  | ***Cell lines*** | |  |
| Missense substitution | KYSE-410 | P29S | Oesopageal carcinoma |
| Missense substitution | NCI-H2085 | P61H | Non-small cell lung carcinoma |
| Missense substitution | LS-411N | C65Y | Adenocarcinoma |
| Missense substitution | HCC-44 | K75N | Non-small cell lung carcinoma |
| Missense substitution | SNG-M | H95R | Endometrial Carcinoma |
| Missense substitution | SLVL | A128V | Splenic B cell lymphoma |
| Nonsense substitution | SNU-1040 | *143* | Colorectal carcinoma |
| Nonsense substitution | SKG-IIIa | C100C | Cervical carcinoma |
| Insertion | KARPAS-45 | ? | Acute lymphoblastic leukaemia |
| **S18-2 protein** | | | |
| ***Tumors*** | | | |
| Synonymous substitution | TCGA-D8-A1Y1-01 | I107I | Breast carcinoma |
| Synonymous substitution | TCGA-EW-A1OY-01 | T90T | Breast carcinoma |
| Synonymous substitution | TCGA-DM-A1DA-01 | **G132G** | Caecum adenocarcinoma |
| Synonymous substitution | TCGA-AY-5543-01 | **G132G** | Colon adenocarcinoma |
| Synonymous substitution | TCGA-BR-6452-01 | A75A | Stomach adenocarcinoma |
| Synonymous substitution | TCGA-BR-A4QL-01 | I134I | Stomach adenocarcinoma |
| Synonymous substitution | TCGA-HU-A4GQ-01 | G229G | Stomach adenocarcinoma |
| Synonymous substitution | TCGA-43-6143-01 | G250G | Lung Carcinoma |
| Missense substitution | TCGA-GM-A3NW-01 | E60K | Breast carcinoma |
| Missense substitution | TCGA-A1-A0SE-01 | R89W | Breast carcinoma |
| Missense substitution | TCGA-AC-A3W6-01 | D110N | Breast carcinoma |
| Missense substitution | TCGA-DM-A28K-01 | **G132C** | Colon adenocarcinoma |
| Missense substitution | TCGA-G4-6315-01 | **G132C** | Colon adenocarcinoma |
| Missense substitution | TCGA-AA-3662-01 | **G132D** | Colon adenocarcinoma |
| Missense substitution | TCGA-25-1313-01 | **G132C** | Ovarian cancer |
| Missense substitution | TCGA-A6-2672-01 | R173W | Colon adenocarcinoma |
| Missense substitution | TCGA-G5-6641-01 | A3G | Rectum Adenocarcinoma |
| Missense substitution | TCGA-AP-A056-01 | S42Y | Endometrial carcinoma |
| Missense substitution | TCGA-AP-A0LF-01 | D116H | Endometrial carcinoma |
| Missense substitution | C0049T | R11M | Kidney Neoplasm |
| Missense substitution | TCGA-B0-5100-01 | L213P | Renal clear cell carcinoma |
| Missense substitution | LC C21 | A2P | Lung Carcinoma |
| Missense substitution | TCGA-91-6829-01 | R173L | Lung Carcinoma |
| Missense substitution | TCGA-78-7159-01 | G229V | Lung Carcinoma |
| Missense substitution | TCGA-25-2391-01 | R149Q | Ovarian cancer |
| Missense substitution | SS6003149 | P209L | Oesophagial carcinoma |
| Missense substitution | PR-3026 | R78C | Prostate carcinoma |
| Missense substitution | cSCCP4 | R20P | Skin squamous cell carcinoma |
| Missense substitution | ME016T | L122F | Skin malignant cancer |
| Unknown | 699 T | ? | Lung Carcinoma |
| Unknown | RK125 C01 | ? | Liver carcinoma |
| ***Cell lines*** | | | |
| Synonymous substitution | NCI-H748 | H23H | Small cell lung carcinoma |
| Synonymous substitution | KYSE-220 | V194V | Oesopageal carcinoma |
| Synonymous substitution | NCI-H1869 | R218Q | Acute lymphoblastic leukaemia |
| Missense substitution | ME-1 | P209P | Ductal carcinoma |
| Mis sense substitution | NCC021 | T242I | Burkitt lymphoma |
| Missense substitution | NAMALWA | H111P | Burkitt lymphoma |
| Missense substitution | ES7 | R215H | Ewings sarcoma |
| Missense substitution | SJSA-1 | R78C | Multipotential sarcoma |
| Missense substitution | SNG-M | R96C | Adenocarcinoma |
| Missense substitution | SK-MES-1 | R218L | Squamous cell lung carcinoma |
| Missense substitution | NCI-H2196 | A3G | Small cell lung carcinoma |
| Missense substitution | EN | N98D | Burkitt lymphoma |
| Missense substitution | JJN-3 | R215C | Leukaemia |
| Missense substitution | KON | S4P | Multipotential sarcoma |
| Missense mutation | MZ7-mel | R158K | Malignant melanoma |
| Nonsense | MOLT-4 | R67* | Acute lymphoblastic leukaemia |
| Frame shift | JHU-022 | H181fs*16 | Acute lymphoblastic leukaemia |
| Unknown | CAMA-1 | ? | Breast carcinoma |
| Unknown | MFM-223 | ? | Ductal carcinoma |
| Unknown | LS-411N | ? | Adenocarcinoma |
| **S18-3 protein** | | | |
| ***Tumors*** | | | |
| Synonymous substitution | TCGA-AG-A002-01 | P141P | Adenocarcinoma |
| Synonymous substitution | TCGA-BP-5175-01 | P133P | Carcinoma |
| Synonymous substitution | TCGA-EB-A551-01 | H112H | Malignant melanoma |
| Synonymous substitution | TARGET-30-PASTKC | V86V | Neuroblastoma |
| Synonymous substitution | TCGA-49-6761-01 | K45K | Carcinoma |
| Missense substitution | HCC97T | Y195C | Liver Carcinoma |
| Missense substitution | HCC97 | Y195C | Liver Carcinoma |
| Missense substitution | 587228 | R173H | Adenocarcinoma |
| Missense substitution | TCGA-D3-A1Q5-06 | P161L | Melanoma |
| Missense substitution | TCGA-A6-6781-01 | P141L | Adenocarcinoma |
| Missense substitution | TCGA-D1-A103-01 | R113C | Endometrial Carcinoma |
| Missense substitution | TCGA-F4-6703-01 | T105A | Adenocarcinoma |
| Missense substitution | HCC159 | G97R | Liver Carcinoma |
| Missense substitution | HCC159T | G97R | Liver Carcinoma |
| Missense substitution | TCGA-A6-5665-01 | R74H | Adenocarcinoma |
| Unknown | 587234 | ? | Adenocarcinoma |
| Unknown | TCGA-B0-5099-01 | G17W | Renal clear cell carcinoma |
| Unknown | HCC136T | M1V | Liver Carcinoma |
| Unknown | ESO-1872 | ? | oesophageal Carcinoma |
| Unknown | HCC136 | M1V | Liver Carcinoma |
| ***Cell lines*** | | | |
| Missense mutation | KARPAS-45 | R173C | Acute lymphoblastic leukaemia |
| Missense mutation | KE-37 | L180V | Acute lymphoblastic leukaemia |
| Missense mutation | NALM-6 | R153H | Acute lymphoblastic B cell leukaemia |
| Missense mutation | OPM-2 | A55V | Plasma cell myeloma |
| Missense mutation | NOS-1 | A55V | NS |
| Missense mutation | LS-411N | V86I | Adenocarcinoma |
| Missense mutation | NUGC-4 | R134W | NS |
| Missense mutation | CP66-MEL | R182K | NS |
| Nonsense substitution | SK-MEL-31 | T41T | NS |
| Unknown | DND-41 | p.? | NS |
| Unknown | SNU-175 | p.? | NS |
