# Supplementary material for: S18 family of mitochondrial ribosomal proteins: evolutionary history and Gly132 polymorphism in colon carcinoma
Source: Oncotarget. 2016 Jul 30;7(34):55649–62. doi: 10.18632/oncotarget.10957 (PMC5342443; doi:10.18632/oncotarget.10957)
Supplement: Supplementary file 4 [file oncotarget-07-55649-s004.docx]

**Table S5.** The samples of colon carcinoma, studied in the present work. All 30 samples were studied. Ten samples for those the PCR products, obtained with forward wild type and mutated primers, were sequenced are indicated in bold.

| **No** | **Age, gender** | **TNM** | **Stage** | **Morphology** |
| --- | --- | --- | --- | --- |
| **1** | **51,m** | **T2N0M0** | **I** | **Adenocarcinoma saucer-shaped** |
| 2 | 61,f | T3N0M0 | II | Adenocarcinoma endophytic |
| 3 | 61,m | T4N0M0 | II | Adenocarcinoma endophytic |
| 4 | 67,m | T4N0M0 | II | Adenocarcinoma endophytic |
| 5 | 54,m | T4N0M0 | II | Adenocarcinoma endophytic |
| **6** | **59,f** | **T4N0M0** | **II** | **Adenocarcinoma exophytic** |
| **7** | **52,f** | **T4N0M0** | **II** | **Adenocarcinoma saucer-shaped** |
| 8 | 51,f | T4N0M0 | II | Combined adenocarcinoma |
| 9 | 50,f | T4N0M0 | II | Combined adenocarcinoma |
| **10** | **66,m** | **T3NXM0** | **II** | **Adenocarcinoma endophytic mucus-producing** |
| 11 | 67,f | T3N0M0 | II | Combined adenocarcinoma |
| 12 | 78,m | T4N0M0 | II | Adenocarcinoma exophytic mucus-producing |
| 13 | 38,f | T4N0M0 | II | Combined adenocarcinoma mucus-producing |
| 14 | 67,f | T3N0M0 | II | Adenocarcinoma exophytic |
| 15 | 55,m | T4N0M0 | II | Adenocarcinoma endophytic |
| 16 | 47,m | T4N0M0 | II | Adenocarcinoma endophytic |
| 17 | 69,m | T4N0M0 | II | Adenocarcinoma endophytic mucus-producing |
| **18** | **60,f** | **T3N0M0** | **II** | **Adenocarcinoma** |
| 19 | 33,f | T3N0M0 | II | Adenocarcinoma endophytic mucus-producing |
| **20** | **52,f** | **T3N1M0** | **III** | **Epidermoid carcinoma** |
| **21** | **63,f** | **T3N1M0** | **III** | **Adenocarcinoma saucer-shaped** |
| 22 | 66,m | T4N2M0 | III | Adenocarcinoma exophytic |
| 23 | 77,m | T3N0M0 | III | Adenocarcinoma endophytic mucus-producing |
| **24** | **58,m** | **-** | **III** | **Adenocarcinoma endophytic** |
| 25 | 67,m | T3N0M0 | III | Glandular carcinoma |
| 26 | 57,f | T4N0M0 | IIIA | Non-epidermoid carcinoma |
| **27** | **80,f** | **T3N1M1** | **IV** | **Adenocarcinoma** |
| 28 | 44,f | T4N0M1 | IV | Adenocarcinoma endophytic |
| **29** | **66,f** | **T4N0M1** | **IV** | **Adenocarcinoma endophytic** |
| 30 | 66,m | T4N0M1 | IV | Adenocarcinoma endophytic |
